# Detection of severe acute respiratory coronavirus virus 2 (SARS-CoV-2) in outpatients: A multicenter comparison of self-collected saline gargle, oral swab, and combined oral–anterior nasal swab to a provider collected nasopharyngeal swab

**DOI:** 10.1017/ice.2021.2

**Published:** 2021-01-13

**Authors:** Christopher E. Kandel, Matthew Young, Mihaela Anca Serbanescu, Jeff E. Powis, David Bulir, James Callahan, Kevin Katz, Janine McCready, Hilary Racher, Elena Sheldrake, Dorothy Quon, Omid Kyle Vojdani, Allison McGeer, Lee W. Goneau, Christie Vermeiren

**Affiliations:** 1 Faculty of Medicine, University of Toronto, Toronto, Ontario, Canada; 2 Michael Garron Hospital, University of Toronto, Toronto, Ontario, Canada; 3 Dynacare Laboratory, Brampton, Canada; 4 Department of Pathology and Molecular Medicine, McMaster University, Hamilton, Ontario, Canada; 5 Research Institute of St Joe’s Hamilton, Hamilton, Ontario, Canada; 6 Department of Laboratory Medicine, University of Toronto, Toronto, Ontario, Canada; 7 Shared Hospital Laboratory, Toronto, Ontario, Canada; 8 North York General Hospital, Toronto, Ontario, Canada; 9 Mount Sinai Hospital, Toronto, Ontario, Canada

## Abstract

**Background:**

Widespread testing for severe acute respiratory coronavirus virus 2 (SARS-CoV-2) is necessary to curb the spread of coronavirus disease 2019 (COVID-19), but testing is undermined when the only option is a nasopharyngeal swab. Self-collected swab techniques can overcome many of the disadvantages of a nasopharyngeal swab, but they require evaluation.

**Methods:**

Three self-collected non-nasopharyngeal swab techniques (saline gargle, oral swab and combined oral-anterior nasal swab) were compared to a nasopharyngeal swab for SARS-CoV-2 detection at multiple COVID-19 assessment centers in Toronto, Canada. The performance characteristics of each test were assessed.

**Results:**

The adjusted sensitivity of the saline gargle was 0.90 (95% CI 0.86-0.94), the oral swab was 0.82 (95% CI, 0.72–0.89) and the combined oral–anterior nasal swab was 0.87 (95% CI, 0.77–0.93) compared to a nasopharyngeal swab, which demonstrated a sensitivity of ˜90% when all positive tests were the reference standard. The median cycle threshold values for the SARS-CoV-2 E-gene for concordant and discordant saline gargle specimens were 17 and 31 (*P* < .001), for the oral swabs these values were 17 and 28 (*P* < .001), and for oral–anterior nasal swabs these values were 18 and 31 (*P* = .007).

**Conclusions:**

Self-collected saline gargle and an oral–anterior nasal swab have a similar sensitivity to a nasopharyngeal swab for the detection of SARS-CoV-2. These alternative collection techniques are cheap and can eliminate barriers to testing, particularly in underserved populations.

Severe acute respiratory syndrome coronavirus 2 (SARS-CoV-2), the virus responsible coronavirus disease 2019 (COVID-19), continues to be a worldwide health crisis. The test, treat, and isolate strategy that has successfully mitigated spread is underpinned by widespread testing.^1^ Currently, the most widely used diagnostic test is real-time reverse transcription polymerase chain reaction (rRT- PCR) on specimens collected from the nasopharynx. This test is uncomfortable and requires travel to a facility where a trained healthcare professional wearing personal protective equipment collects the specimen.^2^ Self-collected specimens can overcome these and other barriers that may limit testing, particularly in disadvantaged groups that have been disproportionately affected by COVID-19.^3^

Of the nasopharyngeal swab–independent techniques evaluated, saliva has been the most widely assessed, with sensitivity ranging from 69.2% to 97.6%.^4^ The main drawbacks of using saliva are the inability of some individuals to produce sufficient quantity of saliva for testing, the challenge of processing specimens in automated systems designed to receive standard swab tubes, the requirement for heat inactivation, and the need for repeat testing of invalid specimens.^5^ These issues may account for some of the variability in performance characteristics observed. Saline gargle and self-collected non-nasopharyngeal swabs can overcome these limitations, but these have not been assessed in outpatients.^6^

We assessed the performance characteristics of self-collected saline gargle, oral swab, and combined oral–anterior nasal swab for the detection of SARS-CoV-2 in 3 outpatient testing centers.

## Methods

### Study population and self-collected specimens

The study population was consecutive individuals presenting to 3 assessment centers in Toronto, Ontario, who had a nasopharyngeal swab obtained for SARS-CoV-2 testing. During 3 separate study periods, a self-collected paired non-nasopharyngeal specimen was requested: saline gargle from August 28 to September 25, 2020; oral swab from September 29 to October 3, 2020; and a combined oral–anterior nasal swab from October 14 to 17, 2020. For the saline gargle, individuals performed a swish and gargle 3 times with 3 mL of 0.9% normal saline. For the oral swab, individuals self-swabbed the back of their tongue, buccal mucosa, then coat the swab in saliva. For the combined oral–anterior nasal swab, each individual self-swabbed the back of their tongue, buccal mucosa, and the anterior aspect of both nares (for complete instructions see the Supplementary Material online). The swab used for the latter 2 techniques was the Miraclean Technology disposable flocked nasal, oral, and throat swab. All specimens were stored at 4°C until testing. This study was approved by the Michael Garron Hospital Research Ethics Board with implied consent for the voluntary self-collection of a second test after the study rationale was explained.

### Study design

Once the nasopharyngeal swab results were reported, all non-nasopharyngeal specimens available from individuals positive for SARS-CoV-2 and a randomly selected sample from individuals who tested negative on the same day (ratio of negative to positive 7–10 to 1) were retrieved for testing. Clinical information (age, sex, and time from symptom onset) for those who were positive by any test was abstracted by chart review from assessments performed by infectious diseases physicians.

### SARS-CoV-2 RNA detection

Nasopharyngeal, oral, and the combined oral–anterior nasal swabs were tested at Shared Hospital Laboratory (Toronto, ON). All swab samples were placed into a guanidine thiocyanate-based transport medium (McMaster Molecular Medium, Bay Area Health Trustee Corporation, Hamilton, Ontario). A 160-µL aliquot was extracted on the MGISP-960 automated workstation using the MGI Easy Magnetic Beads Virus DNA/RNA Extraction Kit (MGI Technologies, Shenzhen, China). Detection of SARS-CoV-2 E-gene and 5’-UTR and the internal control (RNase P), was performed using the Luna Universal Probe One-Step RT-qPCR kit (New England Biolabs, Whitby, Ontario) on the CFX96 Touch Real-time PCR detection system (BioRad, Mississauga, Ontario) as previously described.^7^ A result is considered positive when the cycle threshold (Ct) is below 38 for both targets.

Saline gargle samples were tested at Dynacare Laboratory (Brampton, Ontario) using the ThermoFisher TaqPath COVID-19 Combo Kit (ThermoFisher Scientific, Waltham, MA), with detection of SARS-CoV-2 genes E, S, and ORF1ab conducted using the on real-time PCR system 7500 Fast or QuantStudio 6 (Applied Biosystems, Waltham, MA). Prior to testing, samples were inactivated by heating at 56°C for 30 minutes in a dry bath filled with thermal beads followed by a 30-second vortex. Samples were extracted by combining 200 µL of each patient sample with 250 µL of lysis buffer master mix containing TNA lysis buffer (Omega Bio-tek, Norcoss, GA), Carrier RNA (Omega Bio-tek), and MS2 phage internal control (ThermoFisher). RNA was extracted using MagBind Viral RNA Xpress kit (Omega Bio-tek) on Hamilton Microlab STARlets (Hamilton, Reno, NV). A sample was defined as positive if the viral genome was detected at Ct values of <37 and as negative at Ct values ≥37.

### Statistical analysis

Descriptive statistics were presented as means or medians as appropriate for continuous variables and proportions for categorical variables. Indeterminate test results, in which each of the targets had Ct values >35, were run again and were considered positive for the purposes of analysis if both targets were detected with a Ct below 38. The sensitivity and negative predictive value for nasopharyngeal and the non-nasopharyngeal tests were calculated with the reference standard being the total number of positives by each test being evaluated because no current gold standard exists.^8,9^ The performance characteristics were adjusted for the subsample testing strategy using inverse-probability weighting.^10^ The κ coefficient was used to estimate the agreement between the nasopharyngeal swab and each self-collected technique. The difference in Ct values for the E-gene detected from the standard healthcare provider collected nasopharyngeal swab between the concordant and discordant pairs of each self-collected technique was analyzed using the Wilcoxon rank-sum test. All analyses were performed using R version 4.0.0 software (R Foundation for Statistical Computing, Vienna, Austria).

## Results

Of 19,620 nasopharyngeal swabs performed in the 3 separate study periods (14,491 for saline gargle, 3,542 for oral swab and 1,587 for oral–anterior nose swab), 340 individuals were positive for SARS-CoV-2. Of these 340, 159 (47%) had a paired non-nasopharyngeal sample collected with 64 (33%) of 194 for the saline gargle, 55 (71%) of 77 for the oral swab, and 40 (58%) of 69 for oral–anterior nasal swab. Overall, 608 saline gargles, 605 oral swabs, and 394 oral–anterior nasal swabs were tested. Clinical information was available for 332 (98%) of 340 individuals positive by the nasopharyngeal swab, and 90 (26%) of 340 were asymptomatic at the time of testing. The median age of individuals with COVID-19 was 33 years (IQR, 24–51), 172 (51%) of 340 were female and 16 (10%) of 159 paired specimens were obtained from children (aged <18 years). Those who provided a paired specimen were as likely to be asymptomatic as the group that did not provide a paired specimen (34 of 155 vs 56 of 177; *P* = .06) and had similar time from symptom onset to test: median, 3 (range, 1–5) for paired group and median 2 (range, 0–4) for no paired specimen group (*P* =.14).

The saline gargle was falsely negative in 7 (11%) of 64 samples, the oral swab in 11 (20%) of 55 samples, and the oral–anterior nasal swab in 6 (15%) of 40 samples (Table 1). Unadjusted, the sensitivity of the nasopharyngeal was >90%, whereas the sensitivity of the saline gargle was 0.89 (95% confidence interval [CI], 0.79–0.96), the oral swab was 0.80 (95% CI, 0.68–0.90), and the combined oral–anterior nasal swab 0.86 (95% CI, 0.71–0.95). When accounting for the random sampling of negative specimens and fraction of individuals with a paired non-nasopharyngeal swab, the sensitivity of the nasopharyngeal swab decreased and that of the non-nasopharyngeal techniques increased to 0.90 (95% CI, 0.86–0.94) for the saline gargle, 0.82 (95% CI, 0.72–0.89) for the oral swab, and 0.87 (95% CI, 0.77–0.93) for the oral–anterior nasal swab (Table 2).

**Table 1.** Results of SARS-CoV-2 Detection in Paired Nasopharyngeal Swab and the Non-nasopharyngeal Swab Specimens From Individuals who Presented to an Outpatient COVID-19 Testing Center

Non-nasopharyngeal Swab	Non-nasopharyngeal Swab Result	Nasopharyngeal Swab	
		Positive	Negative
Saline gargle	Positive	57	1
	Negative	7	543
Oral swab	Positive	44	1
	Negative	11	549
Oral–anterior nasal swab	Positive	34	2
	Negative	6	352

**Table 2.** Performance Characteristics of Nasopharyngeal and Non-nasopharyngeal Swab Detection Methods for SARS-CoV-2 by Various Reference Standards^a^

Swab	Reference Standard	Sensitivity^b^	Adjusted Sensitivity^b^	Specificity^b^	NPV^b^	Adjusted NPV^b^	PPV^b^
NP	Gargle + NP	0.98 (0.92–1.00)	0.88 (0.83–0.92)		1.00 (0.99–1.00)	1.00 (0.98–1.00)	
Gargle	Gargle + NP	0.89 (0.79–0.96)	0.90 (0.86–0.94)		0.99 (0.97–0.99)	1.00 (0.98–1.00)	
Gargle	NP	0.89 (0.79–0.96)	0.89 (0.79–0.95)	1.00 (0.99–1.00)	0.99 (0.97–0.99)	1.00 (1.00–1.00)	1.00 (0.94–1.00)
NP	Oral + NP	0.98 (0.90–1.00)	0.92 (0.84–0.97)		1.00 (0.99–1.00)	1.00 (0.95–1.00)	
Oral	Oral + NP	0.80 (0.68–0.90)	0.82 (0.72–0.89)		0.98 (0.97–0.99)	1.00 (0.95–1.00)	
Oral	NP	0.80 (0.67–0.90)	0.80 (0.67–0.89)	1.00 (0.99–1.00)	0.98 (0.97–0.99)	1.00 (1.00–1.00)	0.98 (0.88–1.00)
NP	Oral–Anterior Nasal + NP	0.95 (0.84–0.99)	0.89 (0.80–0.95)		0.99 (0.98–1.00)	1.00 (0.95–1.00)	
Oral–Anterior Nasal	Oral–Anterior Nasal + NP	0.86 (0.71–0.95)	0.87 (0.77–0.93)		0.98 (0.96–0.99)	1.00 (0.95–1.00)	
Oral–Anterior Nasal	NP	0.85 (0.70–0.94)	0.85 (0.70–0.93)	0.99 (0.98–1.00)	0.98 (0.96–0.99)	0.99 (0.99–1.00)	0.94 (0.81–0.99)

Note. NP, nasopharyngeal; NPV, negative predictive value; PPV, positive predictive value.

a Unadjusted and adjusted (by inverse probability weighting) for the testing subsample were calculated.

b Data shown with 95% confidence intervals.

**Fig. 1.**
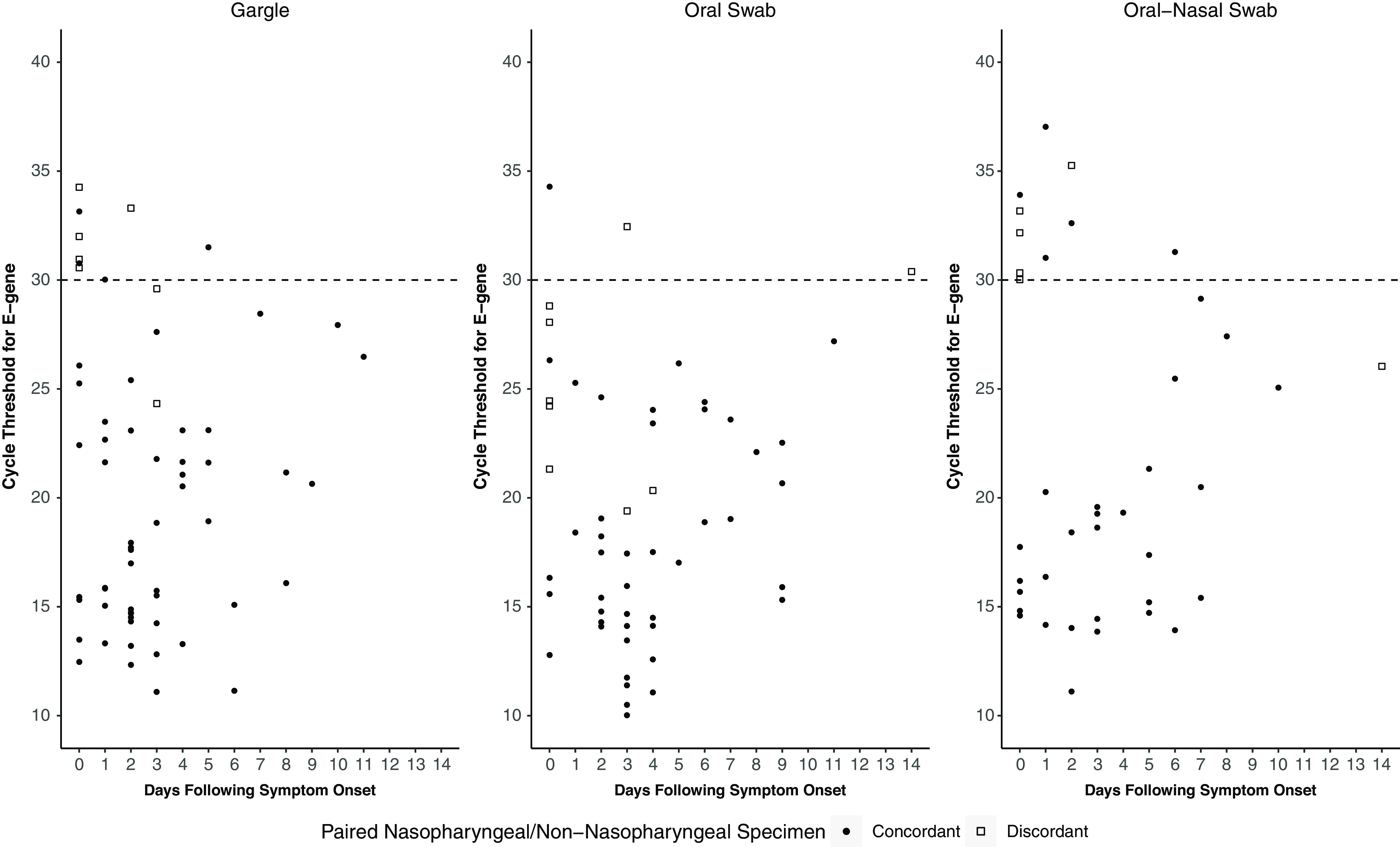
Cycle threshold values of the reverse-transcriptase polymerase chain reaction of the E-gene target of SARS-CoV-2 RNA detected from a nasopharyngeal swab and the result of a paired saline gargle, oral swab or combined oral–anterior nasal swab by time from symptom onset in days (0 indicates those asymptomatic at the time of testing).

The percent agreement between the nasopharyngeal swab and the saline gargle was 0.99 (κ, 0.93; 95% CI, 0.86–0.96), for the oral swab it was 0.98 (κ, 0.87; 95% CI, 0.79–0.92), and for the oral–anterior nasal swab it was 0.97 (κ, 0.85; 95% CI, 0.74–0.91). Discordant saline gargle and oral–anterior nasal swab specimens were observed primarily in asymptomatic individuals with nasopharyngeal specimens that had high Ct values for the E-gene, whereas the oral swab was negative in both symptomatic and asymptomatic individuals (Fig. 1). The median Ct values for nasopharyngeal swab concordant and discordant saline gargle specimens were 17 and 31 (*P* < .001), for the oral swabs these values were 17 and 28 (*P* < .001) and for oral–anterior nasal swabs these values were 18 and 31 (*P* = .007), respectively. The Ct values for those who tested positive in only the saline gargle were 37 for the open-reading-frame target, 33 for the E-gene target for the oral swab, and 25 and 27 for the E-gene for the oral–anterior nasal swabs.

## Discussion

In outpatient COVID-19 testing centers, self-collected techniques are feasible, require minimal instruction, and preclude the presence of a healthcare professional. The combined oral–anterior nasal swab and saline gargle are comparable alternatives to a nasopharyngeal swab, but the lower sensitivity of the oral swab makes this less useful.

Saliva is effective for SARS-CoV-2 detection with an overall sensitivity of 0.91 (95% CI, 0.80–0.99).^4^ The challenge with saliva has been specimen collection and processing, which has spurred the evaluation of oral swabs, which have a sensitivity of ˜90% when a nasopharyngeal swab is the reference standard according to a small study.^11^ Our attempt to increase the sensitivity of oral swabs by maximizing saliva collection was unsuccessful. Using a nylon fiber swab to collect saliva missed individuals with COVID-19 who had Ct values below 24, the concentration at which SARS-CoV-2 can be cultured and theoretically transmitted to others.^12^

Saline gargle can detect SARS-CoV-2 in symptomatic individuals with COVID-19.^6,13^ When evaluated in those with confirmed COVID-19, it was similar to a nasopharyngeal swab with a reported sensitivity of 98%.^6^ This study only tested individuals with prior SARS-CoV-2 detection and who had been symptomatic for days prior to testing. We collected paired nasopharyngeal swab and saline gargle specimens to assess the performance characteristics at the time of diagnosis, the intended use of this technique. Our study population included individuals with short durations of symptoms and those who were asymptomatic, providing a representative sample of the populations who attend COVID-19 testing centers.

Swabbing the anterior nares is less invasive than a nasopharyngeal swab and has a sensitivity of 94.0% (97.5% CI, 83.8–100.0), according to a small study of symptomatic individuals being tested for COVID-19.^11^ We found that swabbing the oropharynx and tongue along with the anterior nares had an adjusted sensitivity approaching 90%, equivalent to a nasopharyngeal swab when all positive specimens were used as the reference standard. Similar to the saline gargle, the oral–anterior nasal swab missed those with high Ct values who were asymptomatic and less like to be infectious.^12^ In addition, the oral–anterior nasal swab identified 2 symptomatic individuals with Ct values of ˜25 who were falsely negative by a nasopharyngeal swab, highlighting the fact that swabbing a second location increases the sensitivity to some degree. Added advantages of the combined oral–anterior nasal swab over the saline gargle are that these tests can be substituted for nasopharyngeal swabs in existing testing platforms, do not require proprietary collection containers, and can be transported in media that inactivates viruses, which obviates the need for heat inactivation.

This study has several limitations. First, saline gargle was tested for SARS-CoV-2 using a different platform than the nasopharyngeal swab, which can be problematic if the analytic thresholds are different. However, in a comparison of 13 different testing platforms, there was minimal difference in the detection limits of SARS-CoV-2.^14^ Second, the proportion of individuals who provided a paired non-nasopharyngeal swab were different in each testing period. Such differences were accounted for using inverse-probability weighting, but this cannot completely overcome self-selection bias whereby individuals who submit a self-collected specimen are systematically different from those who do not. Third, the cohort consisted primarily of adults so the results may not apply to children.

In conclusion, self-collected saline gargle and a combined oral–anterior nasal swab are comparable alternatives to the nasopharyngeal swab in adults presenting to outpatient COVID-19 testing centers. Both techniques are inexpensive, easy to self-collect, reduce exposure of healthcare workers and others to COVID-19, and can be operationalized using the current testing infrastructure. These options may be valuable additions to diagnostic testing, in particular in those who are mandated to undergo repeated testing.

## Supplementary material

For supplementary material accompanying this paper visit https://doi.org/10.1017/ice.2021.2. click here to view supplementary material
