# Supplementary material for: Detection of severe acute respiratory coronavirus virus 2 (SARS-CoV-2) in outpatients: A multicenter comparison of self-collected saline gargle, oral swab, and combined oral–anterior nasal swab to a provider collected nasopharyngeal swab
Source: Infect Control Hosp Epidemiol. 2021 Jan 13:1–5. doi: 10.1017/ice.2021.2 (PMC7870921; doi:10.1017/ice.2021.2)
Supplement: Supplementary file 1 [file S0899823X21000027sup001.docx]

SUPPLEMENTAL MATERIAL for “Detection of SARS-CoV-2 in Outpatients: A Multi-Centre Validation of Self-Collected Saline Gargle, Oral Swab and Combined Oral-Nasal Swab”

APPENDIX 1. Instructions for self-collected saline gargle.

1. Use the orange specimen cup provided to collect your specimen.
2. Open the pink bottle by twisting off the top.
3. Squeeze the bottle so all the salt water goes into your mouth.
4. Rinse your mouth by swishing the liquid back and forth for 5 seconds then gargle like you are using mouthwash for 5 seconds. Repeat this two additional times then spit all the liquid into the orange cup.
5. When finished, replace the orange cap on the container and screw back on tightly.
6. Give bottle to the nurse.

APPENDIX 2. Instructions for the self-collected oral swab.

1. Spend 30-60 seconds pretending to chew and rubbing your cheeks like you are sucking on a hard candy to stimulate production of saliva
2. Remove the top of the collection tube and discard it
3. Remove the swab from its wrapper and grasp the handle
4. Insert the swab into your mouth and rub the swab on the back of your tongue (as far back as you can comfortably reach) in a circular fashion for a few seconds. Then rub the inside of each cheek.
5. Finally, suck on the swab for 5 seconds, swishing saliva around your mouth.
6. Insert the swab into the collection tube and break off the end by bending until it snaps
7. Recap the tube with the red cap and give to the nurse


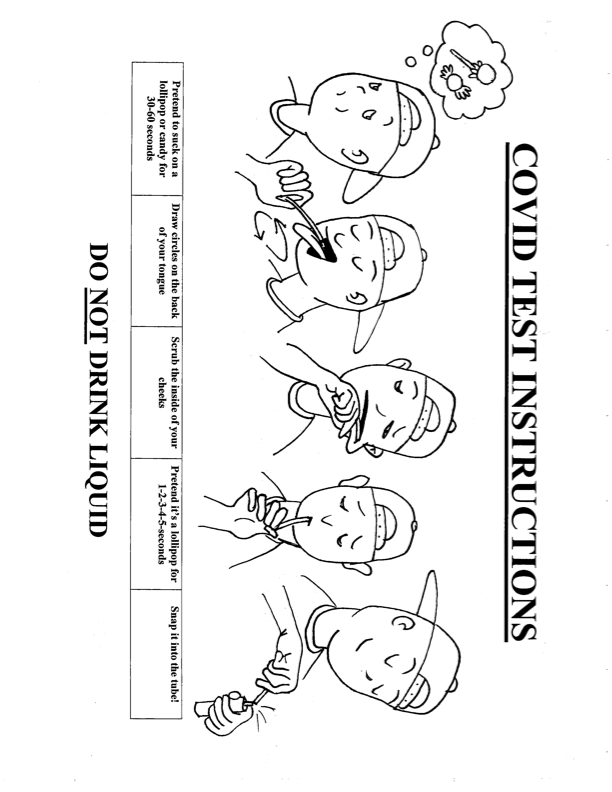


*Courtesy of James Callahan

APPENDIX 3. Instructions for the self-collected oral-nasal swab.

1. Remove swab from wrapper and grasp handle
2. Insert swab into your mouth and rub the back of your tongue (as far back as you can comfortably reach) in a circular fashion for 5 seconds. Then rub the inside of each cheek for 5 seconds.
3. With the same swab insert into the front of one nostril and rub all around three times
4. Repeat in the other nostril
5. Insert swab into collection tube and break off the end by bending until it snaps
6. Put the cap back on the tube and screw it on tightly and return to provider


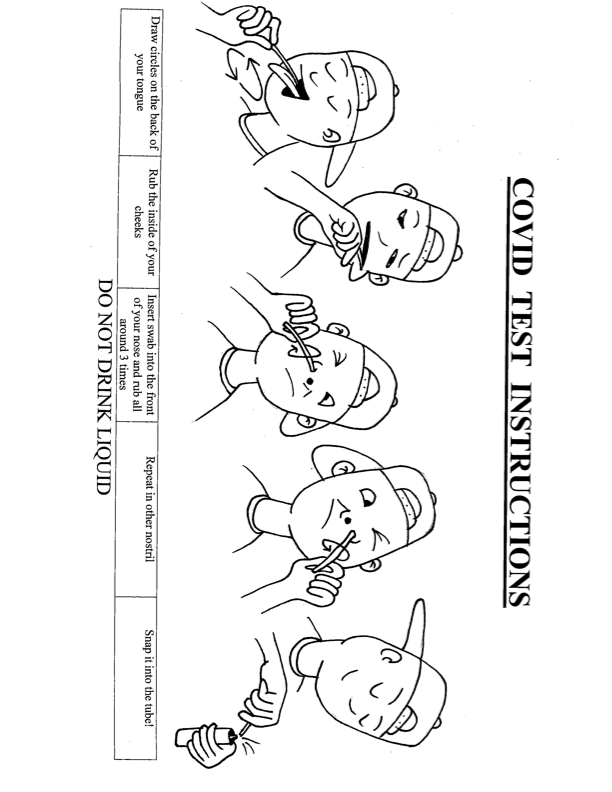


*Courtesy of James Callahan
